# Factors Related with Underperformance in Reading Proficiency, the Case of the Programme for International Student Assessment 2018

**DOI:** 10.3390/ejihpe11030059

**Published:** 2021-07-24

**Authors:** Vianey Vazquez-Lopez, Eric Leonardo Huerta-Manzanilla

**Affiliations:** Graduate School of Engineering, Universidad Autónoma de Querétaro, Querétaro 76010, Mexico; vvazquez31@alumnos.uaq.mx

**Keywords:** supervised classification, logistic regression, PISA 2018

## Abstract

Almost 217 million secondary school students (60% of the world’s adolescents) do not reach minimum levels in reading proficiency at the end of secondary school, according to objective 4.1 of the UN’s Sustainable Development Goals. Therefore, the early and efficient identification of this disadvantage and implementation of remedial strategies is critical for economies. In 2018, the Programme for International Student Assessment (PISA) assessed the reading skills of 15-year-old students in 80 countries and economies. This work introduces a methodology that uses PISA’s data to build logistic regression models to identify the main factors contributing to students’ underperforming reading skills. Results showed that socioeconomic status (SES), metacognition strategies, Information and Communication Technology (ICT) skills, and student–teacher relationships are the most important contributors to low reading abilities.

## 1. Introduction

Around 60% of students globally have a lack of basic reading skills. Of these, the highest proportion is among adolescents. According to UNESCO Institute for Statistics (UIS) data, 61% of adolescents between 12 and 15 years old have not acquired the minimum levels of reading proficiency [1]. This fact occurs even when education coverage is the largest in history [2], and most students who do not reach minimum reading skills attend school. This lack of reading skills indicates that access to education does not guarantee quality learning to solve fundamental problems in reading proficiency [2,3]

This problem has placed the quality of education systems in the focus of governments and international organizations such as the World Bank (WB), which reported 20.6 billion dollars invested in 2576 education programs [4]. The United Nations Educational, Scientific and Cultural Organization (UNESCO) currently has programs dedicated exclusively to improving reading skills [5] and the Organization for Economic Cooperation and Development (OECD), whose research in the area of reading literacy helps to improve education programs worldwide. The initiatives mentioned before align with the United Nations Foundation’s Sustainable Development Goals program.

The Sustainable Development Goals (SDG) is a global plan to mitigate extreme poverty, reduce inequality and protect the planet [6]. There are 17 objectives, of which goal number four is exclusively about quality of education. One of the indicators for this objective is the percentage of students who acquire basic reading skills at the end of their secondary education [7].

Currently, there is no global consensus on the concept of basic reading skills in secondary education, but most organizations agreed to use the definition of the OECD’s Programme for International Student Assessment (PISA) as a reference point [8]. At this level, students begin to demonstrate skills to acquire knowledge and solve practical, real-life problems [8].

The PISA test is one of the best-recognized indexes to know if a student meets the minimum level in reading proficiency. Its first appearance was in 2000, and since then, it has been applied every three years in more than 70 countries. This test measures students’ competence at the end of basic education in reading, mathematics, and science. Reading has been the main topic in the years 2000, 2009, and 2018.

In the area of reading, the PISA test classifies the performance of students in eight levels ranging from 1c, 1b, 1a, 2, 3, 4, 5, 6. 1c is the level with the lowest reading proficiency while level two is the level that defines basic competences and level six is the level that reflects over average competence [8]. Students who obtain a minimum score of 189 are assigned to level 1c; those with a minimum of 698 belong to level six. 

Level two in reading competence is considered the minimum that students must acquire at the end of basic education, around the age of 15; nevertheless, having basic reading skills is insufficient. What is desirable is that students are within or above the OECD average since the development of technology and knowledge worldwide makes it necessary to develop stronger levels of reading ability. In a study by Stuart [9], a project for understanding computational skills versus human literacy skills with data from the OECD Adult Skills Survey, which belongs to the Program for the International Assessment of Adult Competencies (PIAAC), shows that 62% of the participants use PIAAC literacy skills at work, at a level of proficiency that current computers are close to replicating. This result means that soon, technology will replace humans in simple reading tasks since current computers can solve many of the reading problems that students with minimal skills can solve [10].

Governments and international organizations are interested in improving reading literacy due to its impact on individuals’ careers and life prospects, and its effects on economic outcomes. Adolescents must acquire at least the minimum level in reading proficiency. For this, it is necessary to create strategies to improve the reading competence of adolescents. The basis for developing these strategies is knowing the factors that influence the development of reading skills.

The developers of the PISA test understand the importance of knowing these factors; for this reason, in addition to cognitive competence, aspects related to the social, economic, cultural status, well-being, metacognition strategies, and technological factors are evaluated [11]. All the information collected by PISA is stored in a public domain database [12]. These data constitute one of the largest and most complete sources for educational research. The diversity of the questionnaires included in the PISA test enquires into the factors influencing student performance feasible.

The goal of this work was to identify critical factors contributing to reading skills development in school-age students. Based on previous literature, the hypothesis is that factors such as socioeconomic status (SES), metacognition strategies, the use of Information and Communication Technology (ICT), teacher–student relationships, and reading enjoyment/motivation/engagement may predict reading skill achievement. This work introduces a methodology that uses PISA’s data to build logistic regression models to identify the main factors contributing to students’ underperforming reading skills. A supervised classification model based on logistic regression was used to find factors that predict whether a student will score above or below the minimum level in reading proficiency. This research contributes to the generation of knowledge about the development of reading skills. Knowing what factors affect this process can contribute to policymakers and world organizations to improve the teaching process. In addition, the use of classification algorithms demonstrates the usefulness of this type of tool in educational research.

One of the factors mentioned in the literature is SES. The definition of socioeconomic status by The American Psychological Association (APA) is: “the social standing or class of an individual or group” [13]. International large-scale assessments such as PISA, Progress in International Reading Literacy Study (PIRLS) and Trends in International Mathematics and Science Study (TIMMS) report socioeconomic status based on parents’ education, possessions at home, and the number of books at home [14]. Many international large-scale assessments have analyzed the effect of socioeconomic status (SES) on academic performance [15,16,17,18]. In general, weak SES background is associated with academic underperformance [19,20]. However, the cognitive proxies between SES and academic achievement have been explained little; aspects such as executive function [21], peer-effect [22], brain morphometry [23], and other potential links between SES and academic performance have been explored. Still, the understanding of how SES impacts academic development is not well known. Wößmann [24] confirmed that SES (the author’s term was family background) and educational policies such as teaching autonomy and homework were related to better academic outcomes in Korea and Singapore, while Hong Kong and Thailand show less significance.

Another factor reported as a predictor of reading performance is metacognition, a psychological process related to thinking strategies that contribute to better learning and problem solving [25]. According to the OECD, the definition of metacognition is: “a second or higher-order thinking process which involves active control over cognitive processes” [26]. Metacognitive processes are evaluated [11] since various studies indicate that metacognitive strategies are a strong predictor of reading competence [27,28]. Furthermore, students who learn metacognitive strategies obtain better reading competence results than those who receive traditional instruction [29,30]. Sutiyatno and Sukarno [31] and Muhid et al. [32] reported that metacognitive strategies were correlated with reading achievement positively, although both studies had small samples and lack generalizability, these are examples of how metacognitive strategies impact on reading skills.

The metacognitive strategies evaluated in PISA 2018 are three: summarizing and understanding a text, memorizing, and assessing the quality and credibility of sources included in the texts. The last one was integrated into the 2018 version thanks to the growing importance of technology.

With the arrival of the new century, technology and digital texts became common elements in schools and everyday life. Around 2000 and 2010, the investigation of the relationship between reading and technology took a new turn; in these years, researchers began to ask what would be the effects of some technological factors on academic performance and how technology could favor academic performance [33]. The uses of ICT in the classroom may have a negative impact on academic performance; however, it can help underperforming students, as reported by Gómez and Mediavilla [34]. The work by Lee and Wu [35] found that inner ICT state (positive attitude and confidence in the use of ICT) improves reading literacy; however, the availability of ICT at home negatively impacts it. ICT may impact also literacy in mathematics [36].

In 2000, The PISA test was applied for the first time in the year 2000, and with it began the inquiry on the use adolescents gave to technology; later, in the year 2010, research emerged that related to ICT with the performance of the students in reading. These investigations began to show a relationship between cognitive performance and the use of ICT. However, in those years, the questionnaires of familiarity with ICT were shallow, only questioning about accessibility and self-perception of competence in its use [37,38].

The ICT familiarity questionnaire of the PISA test also evolved and became more in-depth with the evolution of technology. This questionnaire investigates issues related to the use of technology outside and inside the school, time of use, and social networks. In addition, this questionnaire allows a more profound knowledge about the role of ICT in cognitive performance.

Some studies report that interest in ICT [39], self-perceived competence in ICT use [40], and autonomy in its use positively impact the level of reading competence [36,41,42]. Other authors point out a negative correlation between the frequent use of ICT in schools and students’ mathematics, reading, and science subjects. In addition, this also indicates that the use of ICT for school purposes is positively correlated, and its use for entertainment purposes has a negative impact. According to these studies, ICT is beneficial as long as it is for educational purposes [40,43].

Contrary to research that claims that ICT use for entertainment is detrimental to reading literacy development, some have found that digital entertainment activities such as chatting, posting on websites, and reading emails are positively related to reading skills [44].

Previous studies seem to contradict each other, but as a possible explanation for this phenomenon, some authors state that the impact of ICT in education depends on the area of study and the type of use [43]. A careful evaluation is required to identify which fields and which uses will positively or negatively impact academic performance, since some uses related to ICT may benefit the area of reading but, on the other hand, be detrimental in mathematics [39].

Other factors potentially affecting reading proficiency development are teacher–student relationship [45], reading enjoyment [46], reading motivation and engagement [47], and video game playing [33,48].

## 2. Materials and Methods

### 2.1. Sample Description

The PISA test 2018 database is the data source for this research. The target population of this test is adolescents between 15 years and 3 months and 16 years and 2 months enrolled in an educational institution and in the seventh higher school year. The number of selected students represents 95% or more of the population [49]. The database has 612,004 records of adolescents from 80 countries and economies, although only 51 of them participate in this study.

The criteria for selecting countries were linked to the data availability, as some presented a large amount of missing data, and it was not possible to perform the analyses. Some countries did not answer specific questionnaires such as the ICT familiarity questionnaires, representing empty variables of interest. Therefore, it was impossible to include them in the analysis. For more information on the excluded countries, Appendix A presents the countries excluded from the study and the reasons for exclusion of each one.

### 2.2. Data 

The adaptation of the data consisted of the reduction and selection of variables of interest since the original database has more than 1000 variables and 80 countries and economies. The process of adapting the data table had numerous changes since variables were discarded or added as the investigation progressed. It began with 1207 variables and 80 countries and economies; at the end, just 13 constructs of interest and 51 countries qualified as significant predictors of reading literacy.

This stage aims to identify the variables showing a relationship with the response variable and know the nature of the relationship, which can be positive, negative, or null. The tool for this was the scatter diagrams. The chosen variables showed a positive or negative correlation with the response variable; those that presented a null correlation concerning the response variable were out of the study.

Two potentially important factors are language and gender; however, as the interest of the study was to find significant constructs at the aggregated level, their study was left for future analysis, with those as controls. Standard linear regressions were initially considered, but it was found that the assumption of normality was not met; therefore, a cut-off point for reading proficiency and logistic regression were used.

### 2.3. Description of Variables

Table 1 contains the 13 selected variables. These variables were presented as significant in all the countries analyzed. Although they were the most representative, it is important to mention that not all were significant in all countries. The computed models are combinations of these factors, although not all in the same regression.

The ESCS factor refers to economic, social, and cultural status. The creators of the PISA test calculate this index. The score obtained comes from the combination of financial, social, cultural, and human capital resources. Specifically, the variables used to obtain this index are parental education, parental occupation, and possessions such as cars, a quiet place to study, internet access, and the number of books or other educational resources available in the home [11].

Factors related to metacognition refer to reading strategies used by students. To elaborate on these constructs (UNDREM, METASUM, METASPAM), we used variables that inquired about student’s usefulness of these strategies [11].

UNDREM stands for “understanding and remembering” and refers to the reading strategy of understanding and remembering. METASUM refers to a strategy of generating abstracts; METASPAM is a concept that was added for the 2018 edition and referred to evaluating the quality and credibility of information in a text.

The variables ICTHOME and ICTSCH refer to the availability of Information and Communication Technologies at home and school, respectively. These constructs are composed of variables that quantified the students’ belongings reported by the students who performed the test. The most important is the availability of personal computers and internet access.

Other variables related to technology are HOMESCH and SOIAICT. The first construct (HOMESCH) is composed of variables that measured whether a student uses ICT for educational purposes outside and within the school and variables that measured frequency with this happened. The SOIAICT construct comprises a series of variables that inquired about ICT as a topic of social interaction.

Some of the significant factors are directly related to the relationship between the students and the teachers. ADAPTATIVITY is a construct that arises from variables related to the level of adaptation of the student to reading classes. TEACHINT refers to the level of support that students perceive from the teacher [11]. 

The calculation of the construct JOYREAD includes variables that directly measured the level of enjoyment that a student has concerning reading fiction, informational, educational texts, among others. In addition, variables in which adolescents answer whether the reading was one of their hobbies and their level of interest in reading [11]. 

The PISADIFF variable consists of a single question, which directly inquired about the level of difficulty of the PISA test that each student had perceived.

Each of the constructs comprises questions from the different questionnaires implemented by PISA; the questions are ordinal variables. The response scales range from 1 to 3, 1 to 4, or 1 to 5. PISA previously calculated the constructs used for this research, and it was not necessary to adjust the data.

Finally, the response variable is the score obtained in reading competence by each of the students. This score is an average of the 10 plausible values provided by PISA. The algorithm transforms the obtained average to zero and one. The cut-off point is the lower limit of the basic level in reading proficiency (level two), which is 407 points. Students who scored below 407 points receive zero, and students who scored above receive one.

### 2.4. Model Estimation

The models used to predict whether a student will score above or below the basic reading proficiency level are binary logistic regressions. These regressions belong to the generalized linear models (GLM), and their main characteristic is that the response variable takes values between 0 and 1 with a Bernoulli random distribution. The general form of these models is in Equation (1)

(1) $$y_{i}=E\left(y_{i}\right)+\varepsilon_{i}$$

Where $y_{i}$ is the probability that the characteristic of interest is present or not, $E\left(y_{i}\right)$ is the response function, and $\varepsilon_{i}$ corresponds to the random error. In this type of model, the response is the probability that the variable of interest takes the value of 1. A linear response function cannot represent these types of responses. A logistic response function should be appropriate for this type of problem. This function’s shape is generally an inverted S or S. The mathematical representation of this function is in Equation (2).

(2) $$E\left(y\right)=\frac{1}{1+exp\left(-x^{\prime }\beta \right)}$$

The estimation method of these models is the one with maximum likelihood, which consists of taking as the estimated value of the parameter of interest that is most likely to occur or best fits the observed data. The main result of the estimations was the significance of the variables for each country. 

#### Odds Ratio 

The essential element of the estimates are the model coefficients (β). In a logistic regression model, to interpret the coefficients the odds ratios should be calculated by computing the exponential of the coefficients. 

After obtaining these values, we proceeded to the forecasting stage. At this point, the algorithm calculates the probability that a student would obtain a score above the minimum level in reading proficiency. The obtained probabilities were transformed into zero and one, assigning a cut-off point equal to 0.5. This cut-off point means that one is for students with 50% or more probability of obtaining an above score; those with a probability less than 50% into zero. 

### 2.5. Validation of the Models

#### ROC Analysis

The ROC curve is a graphical representation that shows all the possible combinations between sensitivity and specificity obtained by a given decision algorithm. It shows the discriminating power of the model to correctly detect positive or negative events.

The basis of this analysis is the confusion matrix, since the Sensitivity (Sen), which is the rates of true positives, and the Specificity (Spe), which is the rate of true negatives, are calculated and plotted. The formulas used for this analysis are Equations (3) and (4).

(3) $$Sen=\frac{TP}{TP+FN}$$

(4) $$Spe=\frac{TN}{TN+FP}$$

*TP* is the positive data (1) correctly classified by the algorithm and *FN* is the number of negatives (0) incorrectly classified by the algorithm. *TN* is the negative data that (0) that were correctly classified by the algorithm, and *FP* is the number of positives (1) correctly classified by the algorithm.

The true positives rate (TPR) is also known as sensitivity, and the false-positive rate (FPR) is 1-specificity. ROC curves have sensitivity on the X-axis and 1-specificity on the Y-axis. Finally, the algorithm calculates the area under the curve (AUC) for each one. The AUC is a probability measure and indicates the probability of correctly classifying the positives and negatives. A perfect model is one with an AUC = 1.

## 3. Results

### 3.1. Predictors by Country

**Figure 1:**
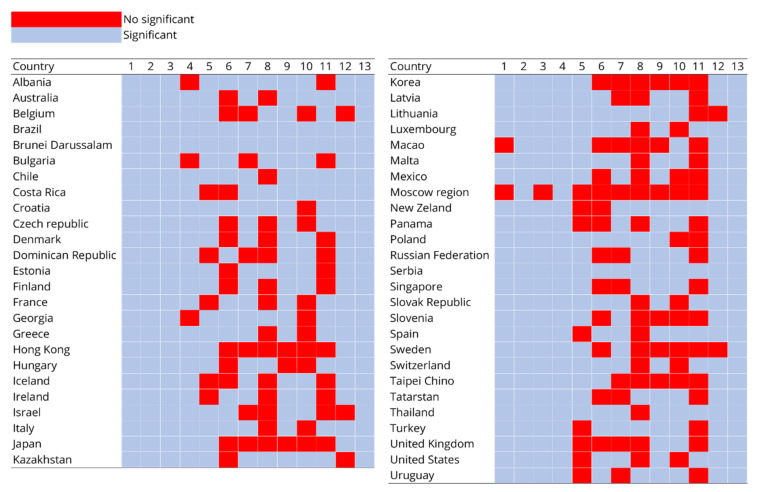
Significant and not significant factor in logistic regressions by country. * ESCS (1), UNDREM (2), METASUM (3), METASPAM (4), ICTHOME (5), ICTSCH (6), HOMESCH (7), SOIAICT (8), DIRINS (9), ADAPTIVITY (10), TEACHINT (11), JOYREAD (12) and PISADIFF (13).

Data analysis resulted in a regression model for each of the 51 countries and economies under study. The participating countries and economies are shown in Figure 1. All the models turned out to be a different combination of the 13 predictors in Table 1.

Figure 1 shows the significant factors for each country as rows. The numbers from 1 to 13 indicate the factors described in Table 2. The blue color indicates a significant factor in the logistic regression under the criterion (*p*-value < 0.05), while those in red were not significant (*p*-value ≥ 0.05). For example, the significant factors for Albania are all except ICTHOME and TEACHINT. The factors that are repeated the most as significant are ESCS, UNDREM, METASUM, METASPAM and PISADIFF. 

### 3.2. Coefficients and Odds Ratio

The coefficients of each of the regressions were transformed into odds ratios (OR) by calculating their exponential. The value of the odds was similar between the countries. Hence, a table was made with the average values to facilitate their interpretation.

The interpretation of odds ratio values in Table 2 is: any value above one indicates a positive correlation between the factor and the response variable. An increase in these indices increases the chances of a student scoring above the minimum level in reading proficiency. Values below one are negatively correlated; that is, an increase in these indices decreases the chances that a student will score above the minimum level in reading proficiency.

The importance of these averages is to know what the range in which the odds ratio related to each factor may be in the 51 countries. Although knowing the exact value of the odds ratios is relevant, it was decided that the table of averages would be used, since the most critical information to communicate with the value of the odds ratio is whether a factor has a positive or negative effect on the development of reading skills, that is if they have an odd ratio above or below than one. This information can be obtained with the average odd ratio and the standard deviation of each of them.

Increasing the ESCS, UNDREM, METASUM, METASPAM, SOIAICT, ADAPTATIVITY, TEACHINT, and JOYREAD factor by one increases the probability that a student will exceed the minimum level in reading proficiency by 76%, 38%, 55%, 55%, 7%, 22%, 18% and 32% respectively. In contrast, an increase of one unit in ICTHOME, ICTSCH, HOMESCH, DIRINS, and PISADIFF decreases the probability that a student will exceed the minimum level in reading proficiency by 11%, 7%, 13%, 19% and 37%, respectively.

### 3.3. Predictions 

The predictions of the logistic regression are, in the first instance, the probabilities that some event of interest will occur. In this case, that a student scores above the minimum level in reading proficiency. The cut-off point for the classification of probabilities is 0.5. Those with less than 50% probability of occurring took zero, while those with a probability greater than 50% took one.

**Figure 2:**
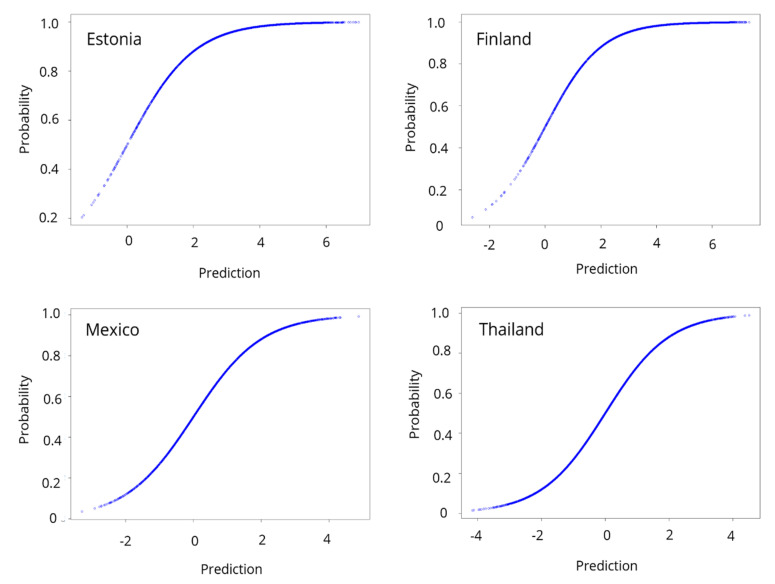
Forecasts from Estonia, Finland, Mexico, and Thailand.

Figure 2 shows some of the graphs of the probabilities obtained with the models. The probability of obtaining a score above the minimum level in reading proficiency is on the Y-axis. In Estonia and Finland, the probability of exceeding the average of the OECD in reading competence accumulates close to one, consistent with reality since these countries obtained scores above the minimum level. In countries such as Mexico and Thailand, the curve shows a very different probability distribution to Estonia and Finland, since the probabilities are distributed more homogeneously and not with a clear tendency towards one, consistently, as these countries scored below the average. 

The graphs of the probabilities show a similar pattern in all countries; the algorithm assigns probabilities with a trend of one in the countries that showed better results; for those below-average scores, the probabilities are close to zero.

### 3.4. Validation of the Models

This section includes the results obtained from the confusion matrices and the receiver operating characteristic curves (ROC curves), which are the main validation methods for each of the 51 models.

The confusion matrices are a count of successes and errors that is made based on a comparison between the forecasts made by the model and real data. This tool indicates the exact number of errors and successes of the model’s predictions.

The ROC curves, on the other hand, indicate the probability that the model is correct in the forecast that a student will obtain the minimum level of reading proficiency.

#### 3.4.1. Confusion Matrix

The results in Table 3 are a summary of the confusion matrices of the 51 countries that participated in the study. The column called True Negative is the number of correct classifications of people who obtained a score below 407 points divided by the actual total of people who did not obtain the minimum score in reading literacy. This calculation is also known as specificity and is calculated with Equation (4). Likewise, the True Positive column contains the percentages of correct classifications of people who obtained scores of 407 points onwards divided by the total number of people who reached the minimum reading skills. This calculation is known as sensitivity and is calculated with Equation (3).

The percentages show that the model has higher values of sensitivity than of specificity, that is, it better predicts students who will obtain a score of 407 or higher, those those will obtain the minimum level in reading proficiency. In countries such as Estonia, Finland, Hong Kong, Ireland, Macao and Singapore, the sensitivity percentage reaches 98%.

#### 3.4.2. Receiver Operating Characteristic Curve (ROC)

A ROC curve and AUC were calculated for each country or economy under study. The smallest value of AUC corresponds to Macao, one of the regions belonging to Albania with an AUC = 0.7651, and the largest corresponds to Finland with an AUC = 0.8955. The average AUC for the 51 countries is 0.8340. These values indicate an 83.40% of probability that the models correctly classify the students who will obtain a reading literacy score above the minimum level in reading proficiency and those who will not.

**Figure 3:**
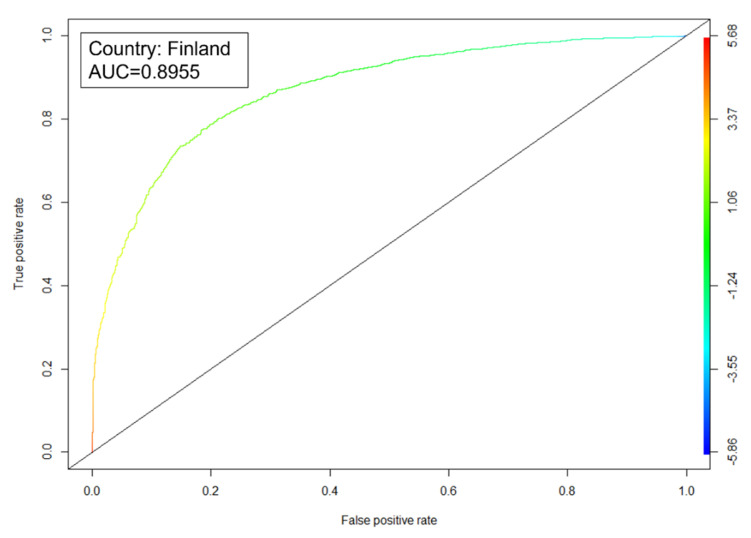
Finland’s ROC curve.

Figure 3 shows an example of the ROC curve obtained for each country. The curve belongs to Finland. The AUC obtained with the algorithm for this country is equal to 0.8703. Finland’s AUC means a probability of 87.03% that the algorithm correctly classifies the students who will obtain a score in reading literacy above the minimum level in reading proficiency and those who will not.

## 4. Discussion

Figure 1 shows the significant factors for each country obtained by the classifier algorithm. Additionally, Table 2 lists the average O.R. of each factor.

The social, economic, and cultural status (ESCS) is a significant predictor in most countries under study, and its average O.R. is close to 2. These data indicate a significant and positive correlation between ESCS and students’ level of reading competence. These results suggest that an increase in the ESCS index increases the probability that students will score above the minimum level in reading proficiency. This result is because ESCS is one of the most significant predictors of academic performance and reading literacy worldwide [20,21,22,23,24]. The ESCS impacts many aspects of students’ characteristics, such as well-being, learning resources, emotional stability, parental support, and even studies that reveal a relationship between ESCS and the brain structure of students [21]. For this reason, the literature indicates that the positive correlation between ESCS and academic performance is a relationship that prevails over time and across nationalities.

The results related to ESCS suggest that national economies may be the frameworks that alter the chances of their citizens to achieve better levels of reading proficiency for those living in developed countries. In contrast, people living in developing countries would be at a disadvantage. Additionally, this may imply that within each country, marginalized families may suffer the same limitations, even living in large economies and well-developed countries. 

In addition to ESCS, the factors related to metacognition (UNDREM, METASUM, and METASPAM) were also significant in most countries and positively correlated with the response variable. The average ORs indicate that the increase in some of the metacognition indices increases the chances of obtaining a score above the minimum level in reading proficiency.

These results are in line with the findings of Sutiyatno and Muhid [31,32], who report a positive correlation between metacognitive strategies and the level of reading competence. The investigations explain that these strategies allow students to organize their thoughts and understand a text in greater depth.

Another factor that was also significant in all countries was the perceived difficulty of the test (PISADIFF). On this occasion, the correlation was negative and this means an increase in the PISADIFF index means a greater probability of obtaining a score below the minimum level in reading proficiency. This finding is common sense, since if a student perceives something as difficult, they are not fully equipped with the skills or abilities necessary to solve the problems he faces.

Other factors that were also significant, but not in all countries, were ICTHOME (ICT available at home), ICTSCH (ICT available at school), HOMESCH (use of ICT outside the education school), and SOIAICT (ICT as a topic of social interaction). Furthermore, these variables showed a negative correlation with the response variable; that is, an increase in the index of these factors means a greater probability that the student will obtain a score below the minimum level in reading proficiency.

These results are similar with those reported in previous studies [23], showing a negative association between HOMESCH and academic performance. In the research by Hu, and Lee and Wu [35,36], they report a negative correlation between ICTHOME and HOMESCH and the level of proficiency in reading. Additionally, Gómez-Fernández and Mediavilla [34] reported a negative effect on academic performance related to the use of ICT at home and school, as well as the importance of ICT as a topic in social interaction. A possible cause that the availability of ICT in the home negatively correlates with ICT use may be the type and quality of use that students give. Students may have developed an addiction to video games [33,48].

In the classification models, variables related to the student–teacher relationship were also significant. These variables are DIRINS (teacher instruction), ADAPTIVITY (adaptation to instruction), and TEACHINT (interest perceived by students by the teacher). These variables were not significant in all countries, but they were in most of them.

The student–teacher relationship is one of the most influential factors in students’ academic performance. The degree of support that students perceive from teachers is an essential factor in the school environment [45]. Students who perceive responsible instruction from the teacher tend to be more persistent and try harder to improve their learning.

Finally, JOYREAD is another factor also significant in most countries. This factor presented a positive correlation with the response variable and means that an increase in this index indicates an increase in the probability that a student will obtain a score above the minimum level in reading proficiency.

Reading enjoyment is an essential part of a student’s motivation to read [46]. The positive relationship between the enjoyment of reading and the level of reading competence found in this research confirms previous studies. With the help of Figure 2, we looked for patterns related to the continent in which the countries are located and their general performance in reading literacy; however, no significant patterns were found. Yet, the validation of the logistic regressions indicates that the factors selected for each country can make proper predictions.

The classifier algorithms for the 51 countries under study are a combination of the factors described in the previous paragraphs. Although these factors mathematically demonstrated significance (*p*-value < 0.05), the research objective is to obtain algorithms capable of correctly classifying the students who will obtain a score above the minimum level and those who will not. 

The ROC curves were the validation tests for each model. On average, these curves showed AUC = 0.834. This result indicates that the models have an average 83.40% probability of correctly classifying the two students. In addition, it means that the significant factors for each country explain in a large percentage the level of reading competence achieved by the students who participated in the study.

Future research is expected to optimize the classifier algorithm and implement several types of algorithms to know which can better fit the database data.

## 5. Conclusions

The algorithms developed for each country predict an average of 87% of the students who obtained a score above the minimum level of reading proficiency (true positives) and 48% of the students who received a score below the minimum level of reading proficiency (true negatives).

The main significant factors common for the 51 countries under study were SES, metacognition strategies, student–teacher relationship, perceived test difficulty, and enjoyment of reading; they were positively related to students’ level of reading competency. Yet, the factors related to the availability and use of technology (ICT skills) were negatively associated with reading competency.

The logistic models have shown an average AUC of 83.40%; this is the probability that the algorithms correctly predict whether a student will obtain a higher or lower score than the minimum level in reading proficiency. The results may be helpful for educational policymakers as an early warning system to identify students at risk of not developing proper reading competence that may harm their potential for completing high school and college education.

The regression models were different for each country; therefore, there was no single typical pattern. Each of the countries participating in the study must pay attention to their respective significant factors. 

Socioeconomic status is significant and increases the probability of improving reading skills; this is further proof that work must continue to eradicate poverty globally and that governments should align with the SDGs. The implications of the results appear to be related to poverty eradication; its impact on reading literacy and its consequences in other aspects of people’s lives cannot be ignored.

Metacognition strategies were also significant in most countries. The statistical results of this research suggest that improving these skills could positively contribute to the achievement of minimum reading skills by adolescents. Emphasis should be placed on teaching adolescents to develop awareness about their thinking that leads them to generate strategies for developing reading skills. Activities as simple as memorizing, summarizing a text, or explaining what was understood from reading can make a big difference.

Finally, the factor related to the perceived difficulty of the PISA test decreases the probability of scoring with 407 points or more by 37%. Based on this, adolescents must be familiar with these tests. There are resources on the OECD’s online page that can help students become familiar with the topics, the complexity of reading and perform adequately on these assessments.

## Figures and Tables

**Table 1:** Description of the variables found as significant in the logistic regressions.

No.	Variable	Description
1	ESCS	Index of economic, social, and cultural status
2	UNDREM	Metacognition: understanding and remembering
3	METASUM	Metacognition: summarizing
4	METASPAM	Metacognition: assessing credibility
5	ICTHOME	ICT available at home
6	ICTSCH	ICT available at school
7	HOMESCH	Use of ICT outside of school for education
8	SOIAICT	ICT as a topic of social interaction
9	DIRINS	Teacher instruction
10	ADAPTIVITY	Adaptation to instruction
11	TEACHINT	Interest perceived by the students on the part of the teacher
12	JOYREAD	Enjoy of reading
13	PISADIFF	Perceived difficulty of the PISA test

**Table 2:** The average value of the Odds ratio of each factor.

No.	Variable	Average Odds Ratio	Standard Deviation
1	ESCS	1.76	0.251
2	UNDREM	1.38	0.151
3	METASUM	1.55	0.156
4	METASPAM	1.55	0.227
5	ICTHOME	0.89	0.050
6	ICTSCH	0.93	0.038
7	HOMESCH	0.87	0.094
8	SOIAICT	1.07	0.153
9	DIRINS	0.81	0.089
10	ADAPTIVITY	1.22	0.137
11	TEACHINT	1.18	0.135
12	JOYREAD	1.32	0.129
13	PISADIFF	0.63	0.075

**Table 3:** Percentages of successes (true positives and true negatives) in the forecasts made by the algorithms.

Country	True Negative (Specificity)	True Positive (Sensitivity)
Albania	81%	60%
Australia	40%	94%
Belgium	35%	95%
Brazil	67%	78%
Brunei	83%	69%
Bulgaria	70%	82%
Chile	54%	90%
Costa Rica	71%	75%
Croatia	33%	93%
Czech republic	34%	95%
Denmark	36%	95%
Dominican Rep.	93%	45%
Estonia	17%	98%
Finland	34%	98%
France	47%	95%
Georgia	84%	62%
Greece	48%	90%
Hong Kong	19%	98%
Hungary	54%	91%
Iceland	45%	90%
Ireland	35%	98%
Israel	45%	91%
Italy	37%	94%
Japan	44%	93%
Kazakhstan	86%	60%
Korea	38%	94%
Latvia	36%	92%
Lithuania	45%	90%
Luxembourg	58%	89%
Macao	12%	98%
Malta	59%	87%
Mexico	70%	73%
Moscow Region	20%	95%
New zealand	47%	94%
Panama	87%	58%
Poland	17%	97%
Russian Federation	31%	95%
Serbia	54%	87%
Singapore	38%	98%
Slovak Republic	47%	88%
Slovenia	38%	92%
Spain	35%	94%
Sweden	27%	96%
Switzerland	49%	92%
Taipei	48%	92%
Tatarstan	41%	91%
Thailand	87%	59%
Turkey	40%	89%
United Kingdom	25%	97%
United States	41%	93%
Uruguay	59%	86%

## Data Availability

The data presented in this study is public and is available on OECD official page: https://www.oecd.org/pisa/data/2018database/.

## Appendix A

Table A1 shows a list of the countries excluded from the study. The main reason for this exclusion is due to some of the variables of interest for this research were not answered in these countries, especially the questionnaire on familiarity with ICT. The lack of any of the 13 variables of interest in any country warranted exclusion from the study.

**Table A1:** Variables of interest missing from the countries excluded from the research.

Country	Missing Variables
United Arab Emirates	ICTHOME, ICTSCH, HOMESCH, SOIAICT
Argentina	ICTHOME, ICTSCH, HOMESCH, SOIAICT
Austria	SOIAICT
Bosnia and Herz.	ICTHOME, ICTSCH, HOMESCH, SOIAICT
Belarus	ICTHOME, ICTSCH, HOMESCH, SOIAICT
Canada	ICTHOME, ICTSCH, DIRINS, TEACHINT, HOMESCH, SOIAICT
Colombia	ICTHOME, ICTSCH, HOMESCH, SOIAICT
Germany	ICTHOME, ICTSCH, HOMESCH
Indonesia	ICTHOME, ICTSCH, HOMESCH, SOIAICT
Jordan	ICTHOME, ICTSCH, HOMESCH, SOIAICT
Kosovo	ICTHOME, ICTSCH, HOMESCH, SOIAICT
Lebanon	UNDREM, METASUM, METASPAM, ICTHOME, ICTSCH, DIRINS, ADAPTIVITY, TEACHINT, JOYREAD, PISADIFF, HOMESCH, SOIAICT
Morocco	PISADIFF
Moldova	UNDREM, METASUM, METASPAM, ICTHOME, ICTSCH, DIRINS, ADAPTIVITY, TEACHINT, JOYREAD, PISADIFF, HOMESCH, SOIAICT
North Macedonia	UNDREM, METASUM, METASPAM, ICTHOME, ICTSCH, DIRINS, ADAPTIVITY, TEACHINT, JOYREAD, PISADIFF, HOMESCH, SOIAICT
Montenegro	ICTHOME, ICTSCH, HOMESCH, SOIAICT
Malaysia	ICTHOME, ICTSCH, HOMESCH, SOIAICT
Netherlands	ICTHOME, ICTSCH, HOMESCH, SOIAICT
Norway	ICTHOME, ICTSCH, HOMESCH, SOIAICT
Peru	ICTHOME, ICTSCH, HOMESCH, SOIAICT
Philippines	ICTHOME, ICTSCH, HOMESCH, SOIAICT
Portugal	ICTHOME, ICTSCH, HOMESCH, SOIAICT
Qatar	ICTHOME, ICTSCH, HOMESCH, SOIAICT
Baku	ICTHOME, ICTSCH, HOMESCH, SOIAICT
B-S-J-Z China	ICTHOME, ICTSCH, HOMESCH, SOIAICT
Cyprus	NO HAY DATOS
Moscow city	NO HAY DATOS
Romania	ICTHOME, ICTSCH, HOMESCH, SOIAICT
Saudi Arabia	ICTHOME, ICTSCH, HOMESCH, SOIAICT

As can be seen in the table, most of the excluded countries did not have the variables related to the availability and use of technology. The variables related to this topic are of high interest for the study and the countries and economies where it was not possible to analyze their influence were discarded from the research.
